# Exonuclease 1 is a Potential Diagnostic and Prognostic Biomarker in Hepatocellular Carcinoma

**DOI:** 10.3389/fmolb.2022.889414

**Published:** 2022-06-13

**Authors:** Jiaxiu Ma, Jiapei Jin, Huishuang Lu, Jin Zhang, Yalan Li, Xuefei Cai

**Affiliations:** The Key Laboratory of Molecular Biology of Infectious Diseases Designated By the Chinese Ministry of Education, Chongqing Medical University, Chongqing, China

**Keywords:** Exo1, hepatocellular carcinoma, clinical outcome, immune cell infiltration, immune checkpoints, DNA methylation, genetic alterations, TP53

## Abstract

**Background:** Hepatocellular carcinoma (HCC) represents a global health challenge. Effective biomarkers are required for an early diagnosis to improve the survival rates of HCC patients. Exonuclease 1 (EXO1) plays a significant role in the DNA repair and recombination mechanisms. This study aimed to investigate the diagnostic and prognostic roles of EXO1 in HCC.

**Methods:** We analyzed the EXO1 expression levels in various cancers including HCC from The Cancer Genome Atlas (TCGA) and Gene Expression Omnibus (GEO) databases. RNA sequencing data were analyzed using the R packages to determine differentially expressed genes (DEGs) between high- and low-EXO1 expressing HCC tissues from the TCGA–LIHC database. A Spearman’s correlation analysis was performed to determine the association between EXO1 expression and immune cell infiltration, and immune checkpoint genes and TP53. MethSurv and CBioPortal databases were used to evaluate the DNA methylation changes and genetic alterations in the *EXO1* gene. A logistic regression analysis was performed to determine the association between EXO1 expression and the clinicopathological characteristics of the HCC patients. The diagnostic and prognostic predictive values of EXO1 were evaluated using the Kaplan–Meier (K-M) survival curves, diagnostic receiver operating characteristic (ROC) curves, nomogram model, and Cox regression analysis.

**Results:** EXO1 expression levels were significantly higher in the tumor tissues and serums of HCC patients compared to the corresponding controls. The DEGs associated with EXO1 were significantly enriched in the cell proliferation pathways. EXO1 expression levels significantly correlated with immune cell infiltration, immune checkpoint genes, and TP53 in the HCC tissues. The DNA methylation status in five CpG islands of the *EXO1* gene was associated with the prognosis of HCC. EXO1 expression levels in the HCC tissues were associated with the tumor grades, alpha-fetoprotein (AFP) levels, and the tumor stages. Cox regression analysis showed that EXO1 was a potential independent risk factor for the overall survival (OS) and disease-specific survival (DSS) of HCC patients. ROC curve analysis showed that EXO1 expression levels accurately distinguished HCC tissues from the adjacent normal liver tissues.

**Conclusion:** Our study demonstrated that EXO1 was a potential diagnostic and prognostic biomarker, and a promising therapeutic target in HCC.

## Introduction

Liver cancer is the sixth most common cancer worldwide and the third leading cause of cancer-related deaths (Torre et al., 2015; Sung et al., 2021). Hepatocellular carcinoma (HCC) accounts for 75%–85% of the total liver cancer cases (Sung et al., 2021). The absence of effective early diagnostic biomarkers is partly responsible for the high mortality rates of HCC patients (Aghoram et al., 2012). Alpha-fetoprotein (AFP) is the only blood test currently available for the non-invasive diagnosis of HCC, but its clinical application is limited by low sensitivity and specificity (Johnson, 2001). Therefore, effective diagnostic biomarkers are urgently needed to accurately detect early HCC.

Exonuclease 1 (EXO1) belongs to the RAD2 nuclease family, which includes FEN1 and XPG (Wilson et al., 1998). EXO1 is a structure-specific endonuclease and exonuclease that plays an important role in DNA replication, telomere maintenance, DNA double strand break repair (DSB), and DNA mismatch repair (MMR) (Tishkoff et al., 1998; Lee and Wilson, 1999; Tomimatsu et al., 2012; Keijzers et al., 2019). EXO1 overexpression is associated with poor prognosis in several cancers (Kretschmer et al., 2011; Muthuswami et al., 2013; de Sousa et al., 2017). Furthermore, a high EXO1 expression in HCC specimens is related to a poor prognosis (Dai et al., 2018).

Currently, the role of EXO1 in tumor immune cell infiltration, immune checkpoints, aberrant DNA methylation and genetic alterations, and diagnosis and prognosis in HCC has not been established. Therefore, in this study, we performed an in-depth bioinformatics analysis to determine the diagnostic and prognostic roles of EXO1 in HCC using the TCGA and GEO databases.

## Materials and Methods

### Data Collection and Ethics Statement

We downloaded the RNAseq data in the Fragments Per Kilobase per Million (FPKM) format and the corresponding clinicopathological information for the 424 HCC patients from the Hepatocellular Carcinoma Project in the TCGA database (https://portal.gdc.cancer.gov/) (TCGA-LIHC). The RNA sequencing data in the FPKM format was converted into the Transcripts Per Million reads (TPM) format. We also downloaded three HCC datasets (GSE46408, GSE84402, and GSE114564) from the GEO database (https://www.ncbi.nlm.nih.gov/geo/). TCGA and GEO databases are publicly available and written informed consent was obtained from the patients prior to data collection.

We randomly selected tumor and adjacent peritumor liver tissues from five HCC patients from the First Affiliated Hospital of Chongqing Medical University. This study strictly followed the principles of medical ethics and was authorized and supervised by the Ethics Committee of Chongqing Medical University (Number: 2021020).

### Bioinformatics Analysis of Exonuclease 1 mRNA Expression Levels in Hepatocellular Carcinoma and Normal Liver Tissue Samples

EXO1 mRNA expression levels were extracted from the TCGA database for 33 human cancers as well as 374 HCC tissues and 50 normal liver tissue samples. Gene expression data were extracted from the GEO database for the GSE46408, GSE84402, and GSE114564 datasets to analyze the EXO1 expression in the HCC samples. The 374 HCC samples were divided into high-EXO1 expression and low-EXO1 expression groups based on the median expression value of EXO1. Differentially expressed genes (DEGs) between the two groups were analyzed using the “DESeq2” (v1.26.0) R package (Love et al., 2014) with log-fold change absolute value >1.5 and *p*-value < 0.05 as the threshold parameters. The volcano plots and heat maps of the DEGs were visualized using the “ggplot2” (v3.3.3) R package.

### Quantitative Real-Time PCR Analysis

Total RNA was extracted from liver tissues of five pathologically diagnosed HCC patients with the TRIzol Universal Reagent (TIANGEN, Beijing, China) according to the manufacturer’s instructions. RNA concentrations were determined on a Nano-500 Micro UV VIS Spectrophotometer (Allsheng, Hangzhou, China). First strand cDNA was synthesized using the Transcriptor First Strand cDNA Synthesis kit (TaKaRa, Otsu, Japan) according to the manufacturer’s instructions. Quantitative real-time PCR (qRT-PCR) was then performed with the SYBR Premix Ex Taq (TaKaRa, Otsu, Japan) on a CFX Connect^TM^ Real-Time System (Bio-Rad, Hercules, CA, United States) and the following primers: EXO1 forward, 5′-TGA​GGA​AGT​ATA​AAG​GGC​AGG​T-3’; EXO1 reverse, 5′-AGT​TTT​TCA​GCA​CAA​GCA​ATA​GC-3’; β-actin forward, 5′-CCT​TCC​TGG​GCA​TGG​AGT​C-3′, and β-actin reverse, 5′-TGA​TCT​TCA​TTG​TGC​TGG​GTG-3’. Each sample was assessed thrice. The relative EXO1 mRNA levels were determined using the 2^-∆∆Ct^ method.

### Functional Enrichment Analysis of Exonuclease 1-Associated Differentially Expressed Genes in Hepatocellular Carcinoma

The “org.Hs.eg.db” (v3.10.0) R package was used to convert entrez ID to the gene symbol. The “ClusterProfiler” (v3.14.3) R package (Yu et al., 2012) was used for the functional annotation and Gene Set Enrichment Analysis (GSEA) of the DEGs. The curated reference genesets from the MgDB file: c2. cp.v7.2. symbols.gmt were selected for GSEA (Subramanian et al., 2005). We identified 569 significantly enriched clusters based on false discovery rate (FDR) <0.25 and *p* (adjust) <0.05 as threshold parameters. A protein–protein interaction (PPI) analysis was performed using the STRING database (Szklarczyk et al., 2019) and visualized using the Cytoscape software (v3.9.0) (Shannon et al., 2003).

### Correlation Analysis Between Exonuclease 1 Expression Levels, Immune Cell Infiltration, and Immune Cell Markers in Hepatocellular Carcinoma

The ssGSEA algorithm in the “GSVA” (v1.34.0) R package (Hanzelmann et al., 2013) was used to evaluate the tumor infiltration status of 24 immune cell types (Bindea et al., 2013), including neutrophils, cytotoxic cells, dendritic cells (DCs), CD8^+^ T cells, plasmacytoid DC (pDC), natural killer (NK) cells, mast cells, T gamma delta (Tgd), type 17 Th (Th17) cells, immature DCs (iDCs), eosinophils, NK CD56^dim^ cells, regulatory T cells (TReg), T effector memory (Tem), T cells, T central memory (Tcm), B cells, type 1 Th (Th1) cells, macrophages, NK CD56^bright^ cells, activated DC (aDC), T follicular helper (TFH), T helper cells, and type 2 Th (Th2) cells. The Spearman’s correlation analysis was performed to determine the relationship between EXO1 expression levels, the immune cell infiltration status, and the immune cell markers.

### Correlation Analysis Between Exonuclease 1 Expression Levels, Immune Checkpoints, and TP53 in Hepatocellular Carcinoma

The association between the EXO1 expression levels, immune checkpoint genes (including CTLA4 and PDCD-1), and TP53 were analyzed in the HCC samples from the TCGA database using the Spearman’s correlation analysis with the “ggplot2” (v3.3.3) R package. The correlation was considered significant with *p* < 0.05 as the threshold.

### Analysis of DNA Methylation Status in the CpG Islands of the Exonuclease 1 Gene

DNA methylation status in the CpG sites of the *EXO1* gene was analyzed in the HCC–TCGA datasets using the MethSurv database (https://biit.cs.ut.ee/methsurv/). Furthermore, the prognostic value of the CpG methylation status of *EXO1* was evaluated in the HCC samples. Moreover, the association between CpG methylation status of EXO1 and overall survival (OS) of HCC was also evaluated.

### Genetic Alterations in the Hepatocellular Carcinoma Samples

The genomic alterations in the *EXO1* gene were analyzed using the cBioPortal (https://www.cbioportal.org/) (Cerami et al., 2012; Gao et al., 2013) in the following three HCC datasets: MSK, Clin Cancer Res 2018 (Harding et al., 2019); AMC Hepatology 2014 (Ahn et al., 2014); and TCGA, Firehose Legacy (https://gdac.broadinstitute.org/runs/stddata__2016_01_28/data/LIHC/20160128/). K–M survival curve analysis and log-rank test was performed to determine the prognostic significance of genomic alterations in the *EXO1* gene. *p* < 0.05 was considered statistically significant.

### Correlation Analysis Between Exonuclease 1 Expression Levels and Clinicopathological Characteristics of Hepatocellular Carcinoma Patients

The clinicopathological data of the HCC patients including overall survival (OS), disease-specific survival (DSS), and progression-free interval (PFI) were extracted from the TCGA–LIHC project and a previously published study of HCC patients (Liu et al., 2018). The differences in various clinicopathological parameters such as race, AFP levels, DSS events, OS events, T stage, tumor status, histological grade, and pathological stage were compared using the R package between the high- and low-EXO1 expression groups. Differences between the groups were analyzed by the Shapiro–Wilk normality test (*p* < 0.05) for data with normal distribution, Kruskal–Wallis Test, and Dunn's multiple hypothesis test. The results for the significance level were corrected by the Bonferroni method. The statistical data were visualized using the “ggplot2” (v3.3.3) R package. The logistic regression analysis was used to evaluate the relationship between EXO1 expression levels and the clinicopathological characteristics of HCC patients.

### Evaluation of the Prognostic Significance of Exonuclease 1 Expression in Hepatocellular Carcinoma

The survival data of HCC patients from the TCGA–LIHC project and previously published data (Liu et al., 2018) were analyzed using the “survival” (v3.2-10) R package (statistical analysis of survival data) and “survminer” (v.0.4.9) R package (visualization) for the prognostic analysis. K–M survival curve analysis as well as univariate and multivariate Cox regression analyses was performed to determine the survival outcomes of HCC patients based on the EXO1 expression levels. The diagnostic ROC curve, time-dependent survival ROC curve, and nomogram model analysis were performed using the “pROC” (v1.17.0.1), “timeROC” (v0.4) (statistical analysis) and “ggplot2” (v3.3.3) (visualization) R packages to evaluate the predictive value of EXO1 expression levels in HCC diagnosis. K–M survival curves were used for the prognostic analysis of the HCC patient subgroups. The results included a collated sample size (percentage), hazard ratio (HR), confidence interval (CI), and *p* values. Forest plots were constructed using the “ggplot2” (v3.3.3) R package.

## Results

### Exonuclease 1 Expression Levels are Significantly Elevated in Multiple Cancers Including Hepatocellular Carcinoma

**FIGURE 1:**
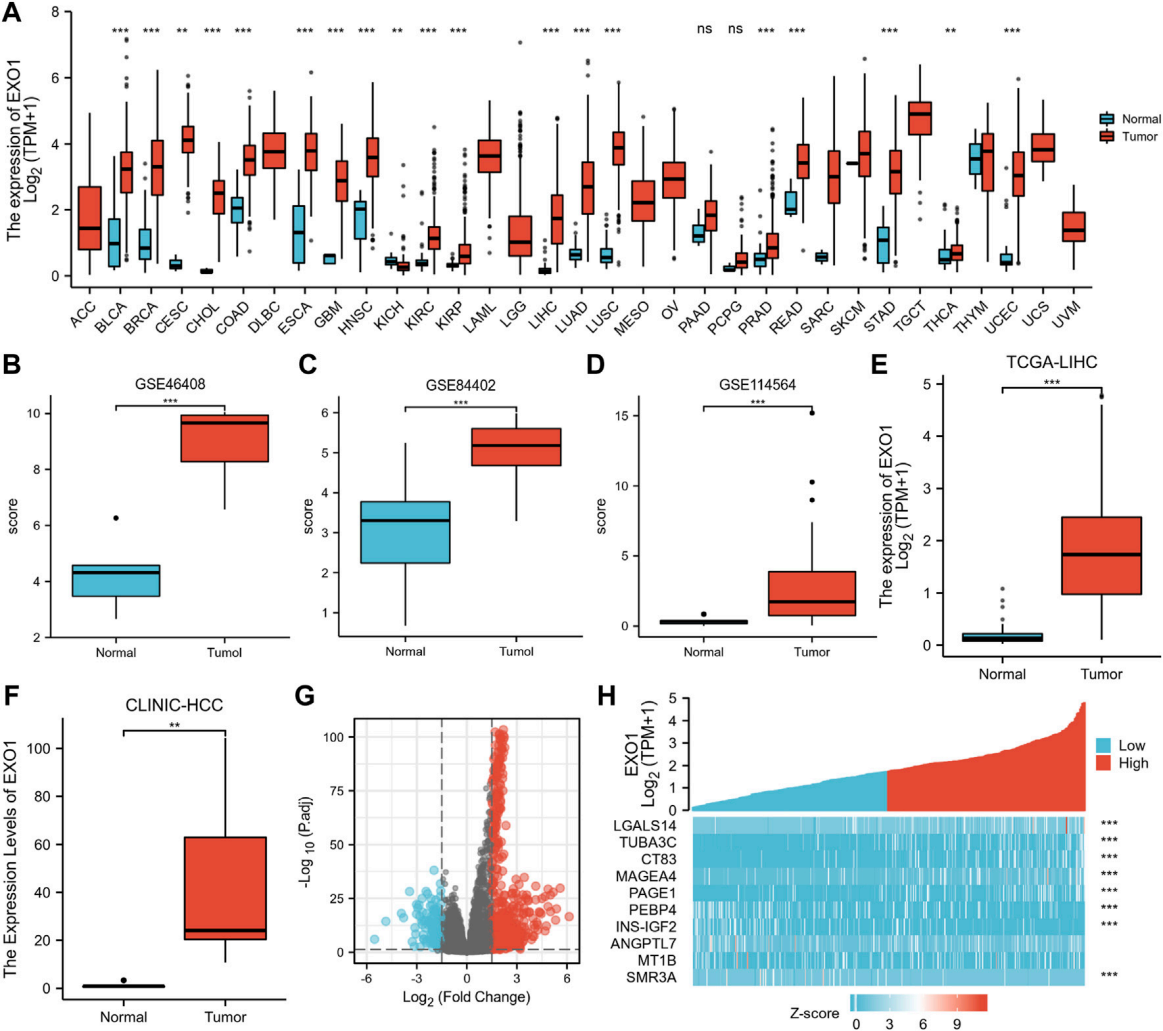
EXO1 expression significantly up-regulated in multiple cancers including HCC. **(A)** TCGA database analysis shows the EXO1 expression levels in 33 types of cancer tissues and their corresponding adjacent normal tissues. ns, *p* ≥ 0.05; **p < 0.01; ***, *p* < 0.001. **(B,C)** EXO1 expression levels were significantly higher in the HCC tissues compared to the adjacent peritumoral liver tissues in the **(B)** GSE46408 and **(C)** GSE84402. **(D)** EXO1 expression levels were significantly higher in the serum of HCC patients compared to the healthy subjects in the GSE114564 dataset (p < 0.001). **(E,F)** EXO1 expression levels were significantly higher in the HCC tissues compared to the adjacent peritumoral liver tissues **(E)** TCGA-LIHC datasets and **(F)** clinical HCC samples. **(G,H)** According to the median EXO1 level, 424 HCC patients from the TCGA–LIHC project were divided into high- and low-EXO1 expression groups. **(G)** The volcano plots and **(H)** the heat maps show the expression levels of specific mRNAs in the HCC patients with high- and low-EXO1 expression (*n* = 422) from the TCGA-LIHC project.

EXO1 expression was analyzed in 33 cancer datasets from the TCGA database. EXO1 was significantly up-regulated in 19 types of cancer tissues out of the 33 (Figure 1A). None of the cancers analyzed showed any significant down-regulation of EXO1. Furthermore, EXO1 expression was significantly higher (*p* < 0.001) in the HCC tissues from the GSE46408 (Figure 1B) and the GSE84402 (Figure 1C) datasets and in the serum of HCC patients from the GSE114564 dataset (Figure 1D) compared to the corresponding controls. Furthermore, the EXO1 expression was significantly higher in the HCC tissues compared to the adjacent peritumoral liver tissues (*p* < 0.001, Figures 1E,F).

### Differentially Expressed Genes Between High- and Low-Exonuclease 1 Expressing Hepatocellular Carcinoma Patients

The median EXO1 expression value was used to classify 424 HCC patients into high- and low-EXO1 expression groups. Then, we used absolute log-fold change >1.5 and *p* < 0.05 as the threshold parameters and identified 794 differentially expressed genes (DEGs) (659 up-regulated and 135 down-regulated) in the high-EXO1 expression group compared to the low-EXO1 expression group (Figure 1G). The six most significant DEGs are shown in the single gene co-expression heat map in Figure 1H.

### Functional Enrichment Analysis of Exonuclease 1-Associated Differentially Expressed Genes in Hepatocellular Carcinoma

**FIGURE 2:**
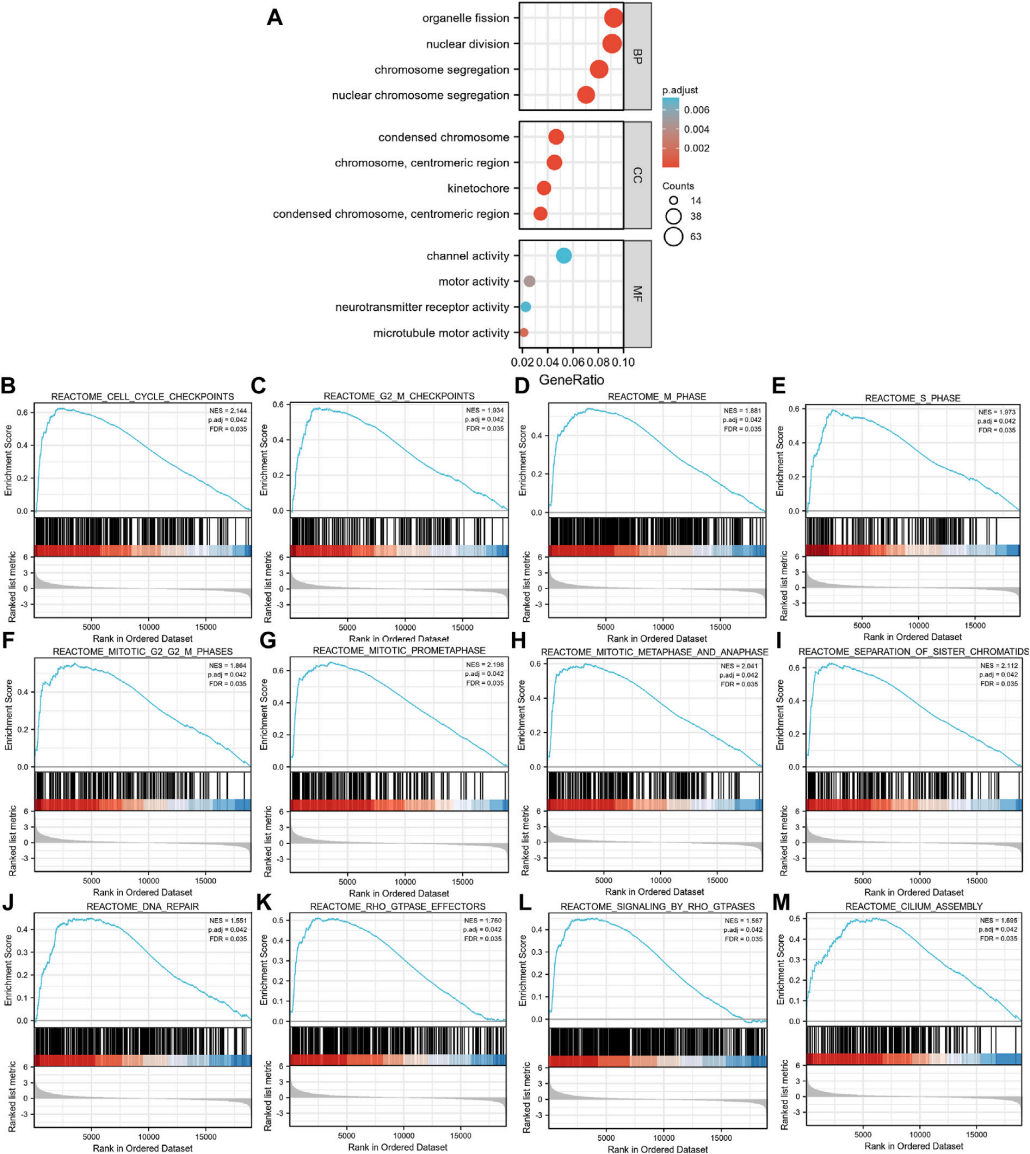
Functional enrichment analysis of the differentially expressed genes (DEGs) based on the EXO1 expression levels in HCC. **(A)** GO enrichment analysis of the EXO1-associated DEGs show the enriched biological functions (BP), cellular components (CC), and molecular functions (MF). **(B–M)** Gene Set Enrichment Analysis (GSEA) of the altered signaling pathways in the HCC tissues based on the EXO1-associated DEGs between the high- and low-EXO1 expression groups in HCC.

We then performed a functional annotation of the EXO1-associated DEGs in the HCC patients using the “clusterProfiler” R package. The GO enrichment analysis results, including the highly enriched biological processes, cellular components, and molecular functions (*p* < 0.05) are shown in Figure 2A and Supplementary Table S1. The top biological processes included “organelle fission,” “nuclear division,” “chromosome segregation,” and “nuclear chromosome segregation.” The most enriched cellular components were “condensed chromosome,” “chromosome”, “centromeric region,” “kinetochore,” “condensed chromosomes”, and “centromeric region.” The most enriched molecular functions were “channel activity,” “motor activity,” “neurotransmitter receptor activity,” and “microtubule motor activity.” GSEA showed that the EXO1-associated DEGs were significantly enriched in cell proliferation-related clusters (Figures 2B–I) including genes related to cell cycle checkpoint (NES = 2.144, Padj = 0.042, FDR = 0.035), G2-M checkpoint (NES = 1.934, Padj = 0.042, FDR = 0.035), M phase (NES = 1.881, Padj = 0.042, FDR = 0.035), S phase (NES = 1.973, Padj = 0.042, FDR = 0.035), mitotic G2-M phase (NES = 1.864, Padj = 0.042, FDR = 0.035), mitotic prometaphase (NES = 2.198, Padj = 0.042, FDR = 0.035), mitotic metaphase and anaphase (NES = 2.041, Padj = 0.042, FDR = 0.035), and separation of sister chromosome (NES = 2.112, Padj = 0.042, FDR = 0.035). EXO1-associated DEGs were also enriched in the DNA repair clusters (Figure 2J). EXO1-associated DEGs showed significant correlation with the RHO-GTPASE effectors and signaling pathways regulated by RHO-GTPASE (Figures 2K,L), and the cilium assembly (Figure 2M). The PPI network of the DEGs (Supplementary Figure S1) showed an association between EXO1 and a total of 17 genes.

### Exonuclease 1 Expression Levels Correlate With the Infiltration of Multiple Immune Cell Types in the Hepatocellular Carcinoma Tissues

**FIGURE 3:**
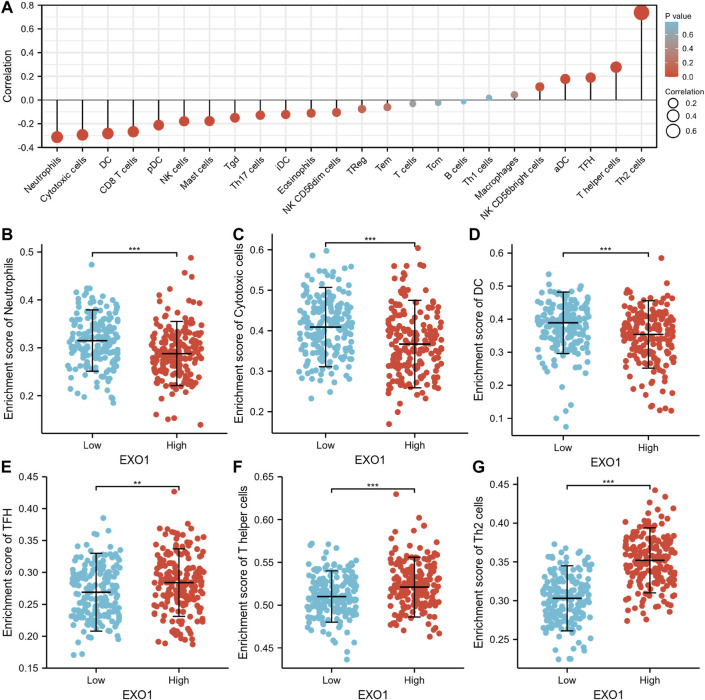
The correlation analysis between immune cell infiltration and EXO1 expression in HCC. **(A)** Spearman’s correlation analysis results between infiltration levels of 24 immune cell types and EXO1 expression levels in the HCC tissues. **(B–G)** The infiltration levels of **(B)** neutrophils, **(C)** cytotoxic cells, **(D)** DC cells, **(E)** TFH, **(F)** T helper cells, and **(G)** Th2 cells in the high- and low-EXO1 expression groups. Note: DC, dendritic cells; pDC, plasmacytoid DC; NK, natural killer cells; Tgd, T gamma delta; Th17, type 17 Th cells; iDCs, immature DCs; TReg, regulatory T cells; Tem, T effector memory; Tcm, T central memory; Th1 cells, type 1 Th cells; aDC, activated DC; TFH, T follicular helper; Th2, type 2 Th cells.

The infiltration status of 24 immune cell types in the HCC tissues was evaluated by ssGSEA. The association between EXO1 expression and immune cell infiltration was estimated by the Spearman’s correlation analysis. EXOI expression levels showed a negative association with neutrophils (R = −0.313, *p* < 0.001), cytotoxic fine cells (R = −0.294, *p* < 0.001), and CD8^+^ T cells (R = −0.267, *p* < 0.001), and positive association with aDCs (r = 0.177, *p* < 0.001), T helper cells (r = 0.277, *p* < 0.001), and type 2 T helper cells (r = 0.739, *p* < 0.001) (Figure 3A). The tumor infiltration levels of neutrophils (Figure 3B), cytotoxic cells (Figure 3C), DC (Figure 3D), TFH (Figure 3E), T helper cells (Figure 3F), and Th2 cells (Figure 3G) were consistent with the Spearman’s analysis results shown in Figure 3A. Next, we evaluated the correlation between EXO1 expression and the immune cell markers. EXO1 expression levels showed positive correlation with specific biomarkers for the B cells (CD19, CD20, and CD38), CD8^*+*^ T cells (CD8A, CD8B), other T cell subsets (TFH, Th2, and Treg), M1 macrophages (IRF5), M2 macrophages (CD115), and TAMs (PDCD1LG2, CD80, and CD40) in the HCC tissues (Supplementary Table S2).

### Exonuclease 1 Expression Levels Correlate With the Expression of Immune Checkpoint Genes and TP53 in the Hepatocellular Carcinoma Tissues

**FIGURE 4:**
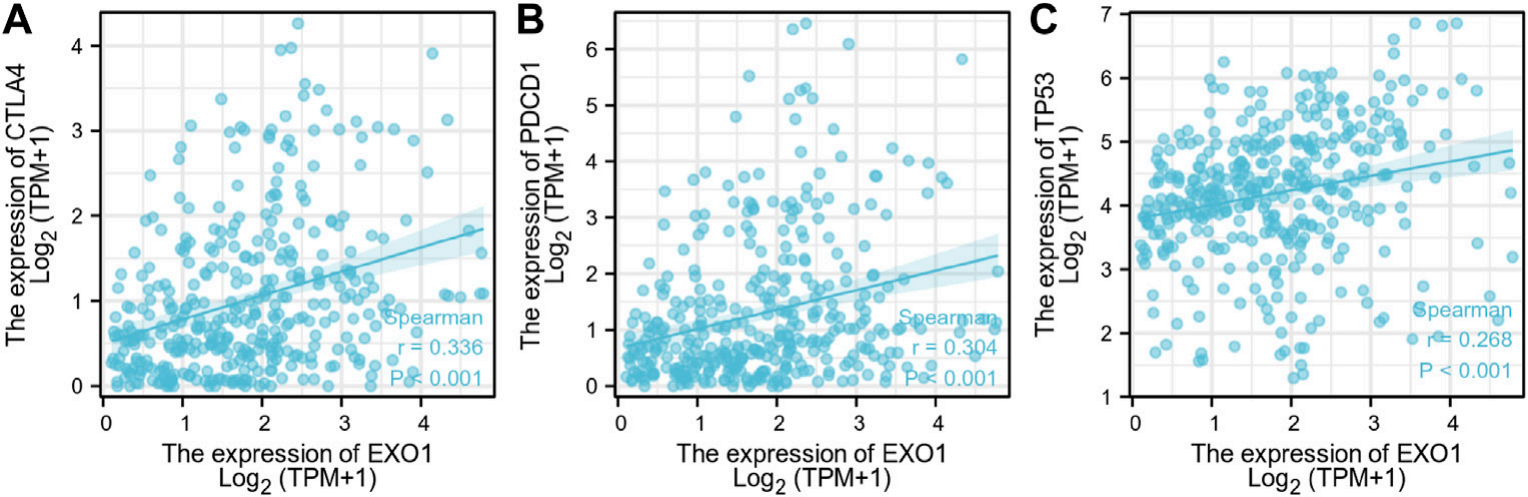
The correlation analysis between the expression levels of EXO1, CTLA-4, PDCD-1, and TP53 in HCC. **(A–C)** The correlation analysis results between the expression levels of EXO1 and the expression levels of **(A)** CTLA-4, **(B)** PDCD-1, and **(C)** TP53 in the TCGA-LIHC dataset.

CTLA-4 and PDCD-1 are important immune checkpoint proteins that are associated with tumor immune escape (Krummel and Allison, 1996; Goodman et al., 2017). Furthermore, *TP53* is a tumor suppressor gene with low expression in the normal cells and high expression in the malignant tumors (Wang and Sun, 2017). We demonstrated that EXO1 expression levels showed positive correlation with the expression levels of CTLA-4, PDCD-1, and TP53 in the HCC samples of the TCGA dataset (Figures 4A–C).

### Methylation Status of the Exonuclease 1 Gene Is Associated With the Prognosis of Hepatocellular Carcinoma Patients

**FIGURE 5:**
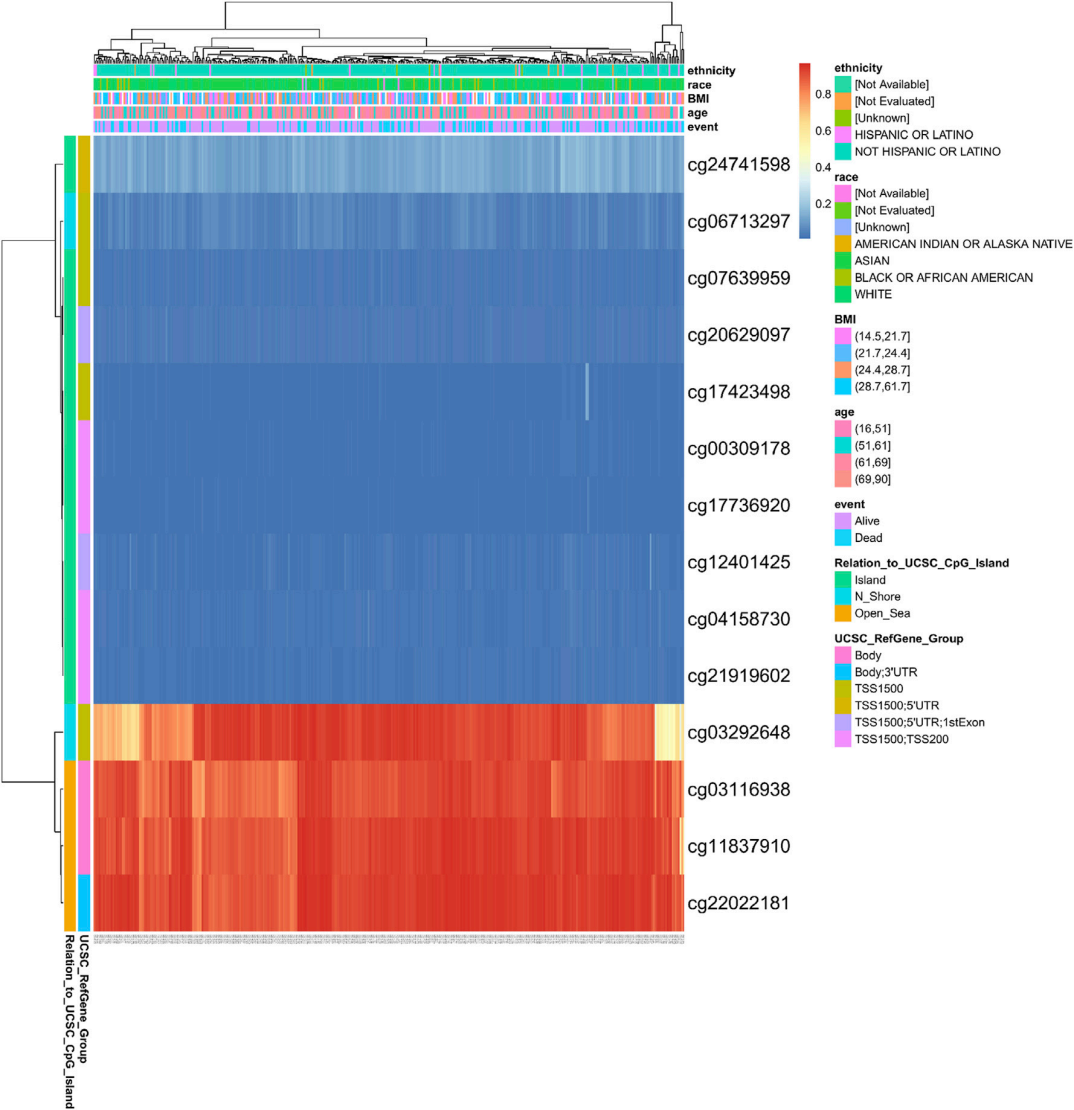
DNA methylation levels in the *EXO1* gene are associated with the prognosis of HCC patients.

DNA methylation levels in the *EXO1* gene and the prognostic value of the CpG islands in the *EXO1* gene were analyzed using the MetSurv tool. The results showed 14 methylated CpG islands including cg03292648, cg03116938, cg11837910, and cg22022181 that showed elevated levels of DNA methylation (Figure 5). Furthermore, methylation levels of six CpG islands, namely, cg03116938, cg03292648, cg11837910, cg21919602, cg22022181, and cg24741598 were associated with the prognosis (*p* < 0.05) (Table 1). Elevated levels of *EXO1* methylation in these six CpG islands, especially cg03292648, were associated with a poorer overall survival of HCC patients compared to those with lower levels of CpG methylation in *EXO1*.

**TABLE 1:** Effects of methylation levels in the CpG sites of the *EXO1* gene on the prognosis of HCC patients.

CpG island	HR	*p*-value
TSS1500; TSS200-Island-cg00309178	1.397	0.11
**Body-Open_Sea-cg03116938**	**2.104**	**0.0023**
**TSS1500-N_Shore-cg03292648**	**0.548**	**0.0007**
TSS1500; TSS200-Island-cg04158730	1.17	0.38
TSS1500-N_Shore-cg06713297	1.163	0.4
TSS1500-Island-cg07639959	0.808	0.29
**Body-Open_Sea-cg11837910**	**1.855**	**0.0025**
TSS1500; 5′UTR; 1stExon-Island-cg12401425	1.458	0.079
TSS1500-Island-cg17423498	1.39	0.06
TSS1500; TSS200-Island-cg17736920	0.832	0.3
TSS1500; 5′UTR; 1stExon-Island-cg20629097	1.147	0.43
**TSS1500; TSS200-Island-cg21919602**	**1.547**	**0.013**
**Body;3′UTR-Open_Sea-cg22022181**	**2.375**	**0.00052**
**TSS1500;5′UTR-Island-cg24741598**	**1.662**	**0.03**

HCC, hepatocellular carcinoma; HR, hazard ratio.

### Genetic Alterations in Exonuclease 1 Are Not Associated With Survival Outcomes in Hepatocellular Carcinoma Patients

**FIGURE 6:**
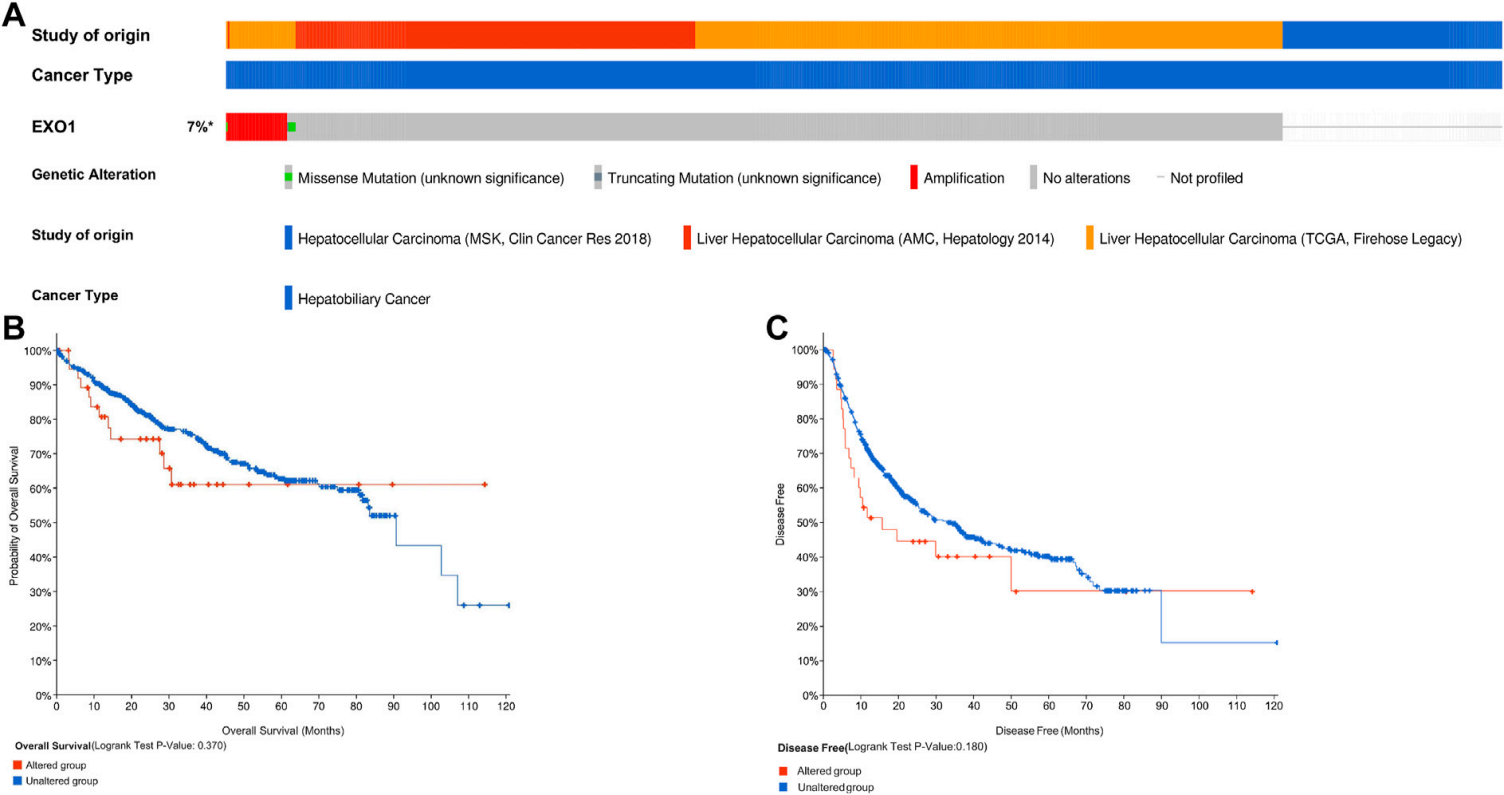
*EXO1* gene alterations are not associated with the survival outcomes in HCC. **(A)** OncoPrint visual summary of the alterations in the *EXO1* gene. **(B,C)** Kaplan–Meier survival curves show the **(B)** overall survival and **(C)** disease-free survival rates of HCC patients with or without *EXO1* gene alterations.

We then analyzed genetic alterations in the *EXO1* gene based on samples from 737 HCC patients from the following three datasets: MSK, Clin Cancer Res 2018 (*n* = 127) (Harding et al., 2019); AMC, Hepatology 2014 (*n* = 231) (Ahn et al., 2014), and TCGA, Firehose Legacy (*n* = 379). Genetic alterations in the *EXO1* gene were observed in only 7% of the HCC patients (Figure 6A). K–M survival curves and a log-rank test showed no significant differences in OS (*p* = 0.370) and DSS (*p* = 0.180) between patients with or without genetic alterations in the *EXO1* gene (Figures 6B,C).

### Exonuclease 1 Expression Levels Correlate With Multiple Clinicopathological Characteristics in Hepatocellular Carcinoma

**FIGURE 7:**
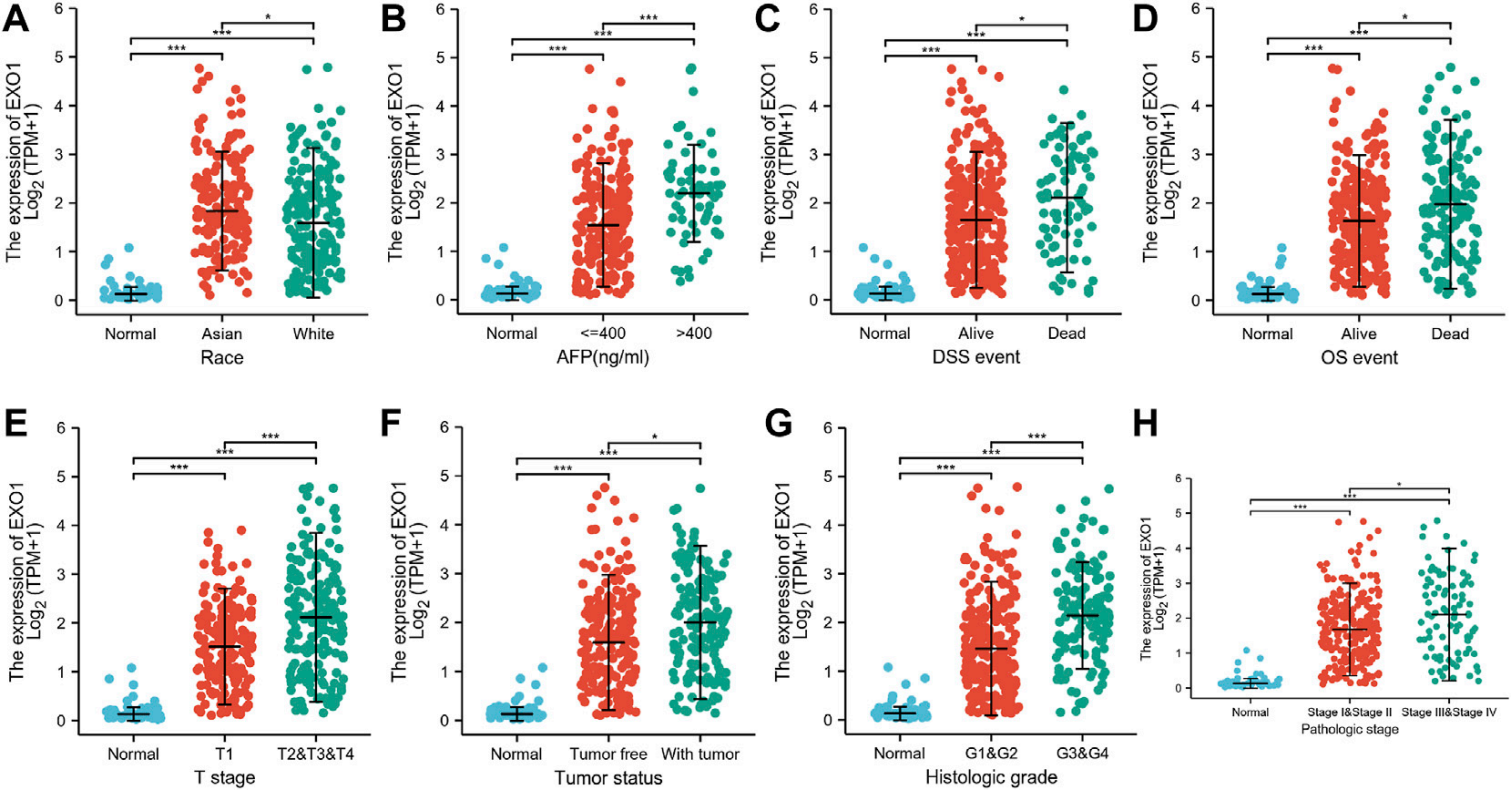
EXO1 expression levels correlate with multiple clinicopathological characteristics of HCC patients. **(A–H)** The correlation analysis between EXO1 expression levels and **(A)** race, **(B)** AFP levels, **(C)** DSS, **(D)** OS, **(E)** T stages, **(F)** tumor status, **(G)** histologic grades, and **(H)** pathologic stages of HCC patients. *p < 0.05, ***p < 0.001.

The association between clinicopathological characteristics of HCC patients and the EXO1 expression levels based on the TCGA–LIHC dataset is shown in Table 2. HCC patients with high- and low-EXO1 expression levels showed significant differences in the clinical T stages, pathological stages, tumor status, histological stages, alpha-fetoprotein (AFP) levels, overall survival (OS), and disease-specific survival (DSS). EXO1 expression levels showed a significant correlation with the race (Figure 7A), AFP levels (Figure 7B), DSS (Figure 7C), OS (Figure 7D), T stages (Figure 7E), tumor status (Figure 7F), histologic grades (Figure 7G), and pathologic stages (Figure 7H) of the HCC patients. Asian patients showed a significantly higher EXO1 expression than the other races. Furthermore, HCC patients with high AFP levels, low OS and DSS rates, and advanced cancer stages also showed higher EXO1 levels. The logistic regression analysis showed that the EXO1 expression levels positively correlated with T stage, tumor status, histologic grade, and AFP levels in the HCC tissues (Table 3).

**TABLE 2:** Clinicopathological characteristics of HCC patients with high- and low-EXO1 expression levels.

Characteristic	Low-EXO1 expression	High-EXO1 expression	*p*
Total number of patients	187	187	
Race, n (%)			0.383
Asian	72 (19.9%)	88 (24.3%)	
Black or African American	8 (2.2%)	9 (2.5%)	
White	97 (26.8%)	88 (24.3%)	
T stage, n (%)			<0.001
T1	112 (30.2%)	71 (19.1%)	
T2	34 (9.2%)	61 (16.4%)	
T3	33 (8.9%)	47 (12.7%)	
T4	5 (1.3%)	8 (2.2%)	
Pathologic stage, n (%)			<0.001
Stage I	105 (30%)	68 (19.4%)	
Stage II	33 (9.4%)	54 (15.4%)	
Stage III	33 (9.4%)	52 (14.9%)	
Stage IV	4 (1.1%)	1 (0.3%)	
Tumor status, n (%)			0.009
Tumor free	114 (32.1%)	88 (24.8%)	
With tumor	64 (18%)	89 (25.1%)	
Histologic grade, n (%)			<0.001
G1	39 (10.6%)	16 (4.3%)	
G2	101 (27.4%)	77 (20.9%)	
G3	41 (11.1%)	83 (22.5%)	
G4	3 (0.8%)	9 (2.4%)	
AFP (ng/ml), n (%)			<0.001
≤400	123 (43.9%)	92 (32.9%)	
>400	21 (7.5%)	44 (15.7%)	
OS events, n (%)			0.007
Alive	135 (36.1%)	109 (29.1%)	
Dead	52 (13.9%)	78 (20.9%)	
DSS events, n (%)			0.005
Alive	155 (42.3%)	132 (36.1%)	
Dead	28 (7.7%)	51 (13.9%)	
Age, median (IQR)	63 (53.5, 70)	60 (51, 68)	0.080

**TABLE 3:** Logistic regression analysis of the relationship between clinicopathological characteristics and the EXO1 expression levels in HCC patients.

Characteristics	Total (N)		*p* value
Race (White vs. Asian)	345	0.742 (0.485–1.134)	0.169
T stage (T2, T3&T4 vs. T1)	371	2.541 (1.678–3.875)	<0.001
Pathologic stage (Stages III and IV vs. Stages I and II)	350	1.620 (1.000–2.646)	0.051
Tumor status (With tumor vs. Tumor free)	355	1.801 (1.180–2.763)	0.007
Histologic grade (G3&G4 vs. G1&G2)	369	3.148 (2.028–4.942)	<0.001
AFP (ng/ml) (>400 vs. ≤400)	280	2.801 (1.576–5.110)	<0.001

HCC, hepatocellular carcinoma; AFP, alpha-fetoprotein.

### Exonuclease 1 Is a Potential Prognostic and Diagnostic Biomarker in Hepatocellular Carcinoma

**FIGURE 8:**
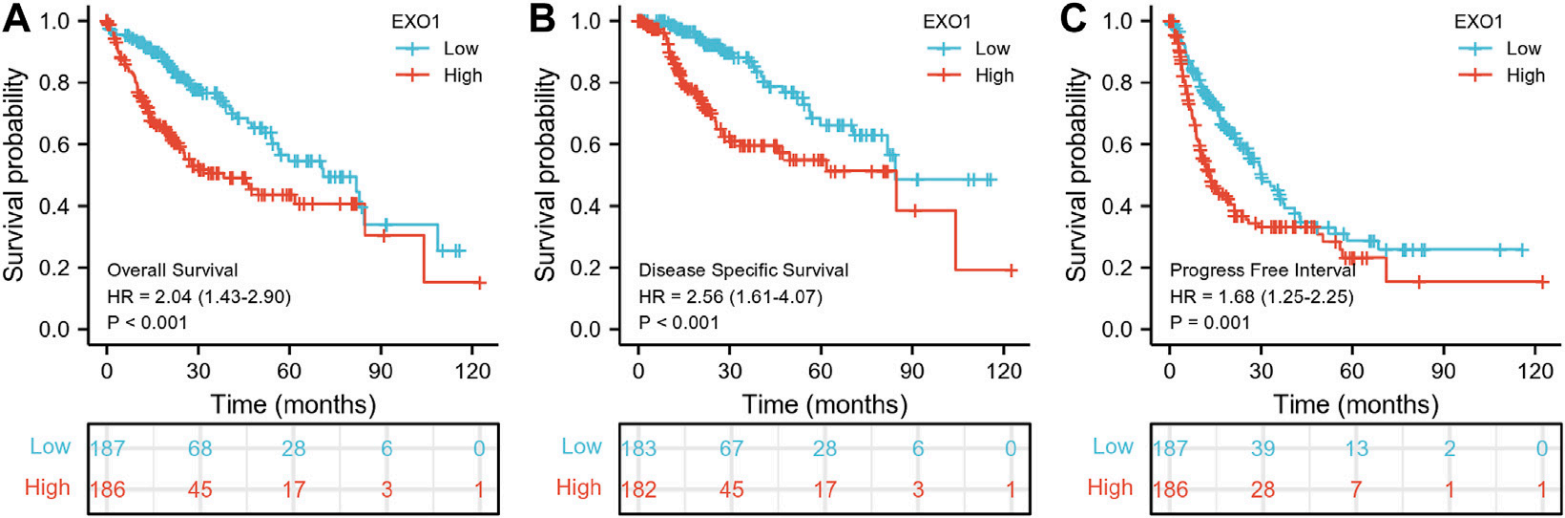
EXO1 shows a high prognostic prediction value in HCC patients. The Kaplan–Meier plotter database analysis shows the differences in **(A)** overall survival, **(B)** disease-specific survival, and **(C)** progression-free interval of HCC patients with high- and low-EXO1 expression levels. *p* < 0.05 indicates statistical significance. The red and blue curves represent high and low EXO1 expressing HCC patients, respectively.

K–M survival curve analysis showed that HCC patients with a high-EXO1 expression were associated with significantly lower OS (*p* < 0.001) (Figure 8A), DSS (*p* < 0.001) (Figure 8B) and progression-free interval (PFI) (*p* = 0.001) compared to those with low-EXOI expression levels (Figure 8C). The multivariate Cox regression analysis showed that EXO1 was an independent risk factor for predicting OS (HR: 1.648, *p* = 0.01) and DSS (HR: 2.216, *p* = 0.003) but not PFI (Table 4). The pathologic stage was an independent predictor of OS and DSS. The tumor status was an independent predictor of OS and PFI. T stages showed a significant clinical value in predicting PFI.

**TABLE 4:** Cox regression analysis of the clinical outcomes in HCC patients based on various clinicopathological characteristics including EXO1 levels.

Characteristics	HR for overall survival (95% CI)		HR for disease-specific survival (95% CI)		HR for progression-free interval (95% CI)	
	Univariate	Multivariate	Univariate	Multivariate	Univariate	Multivariate
Race (Asian vs. White)	1.324		1.544	0.983	1.280	
T stage (T1 vs. T2&T3&T4)	2.126^***^	1.229	2.829^***^	1.229	2.360^***^	1.508^*^
Pathologic stage (Stage I and II vs. Stage III and IV)	2.504^***^	1.776^*^	3.803^***^	2.900^**^	2.201^***^	1.292
Tumor status (Tumor free vs. With tumor)	2.317^***^	1.723^**^	775790759.389		11.342^***^	10.602^***^
Histologic grade (G1&G2 vs. G3&G4)	1.091		1.086		1.152	
AFP (ng/ml) (≤400 vs.> 400)	1.075		0.867		1.045	
EXO1 (Low vs. High)	2.036^***^	1.684^**^	2.564^***^	2.216^**^	1.678^***^	1.200

HCC, hepatocellular carcinoma; HR, hazard ratio; CI, confidence interval; AFP, alpha-fetoprotein; *p < 0.05; **p < 0.01; ***p < 0.001.

**FIGURE 9:**
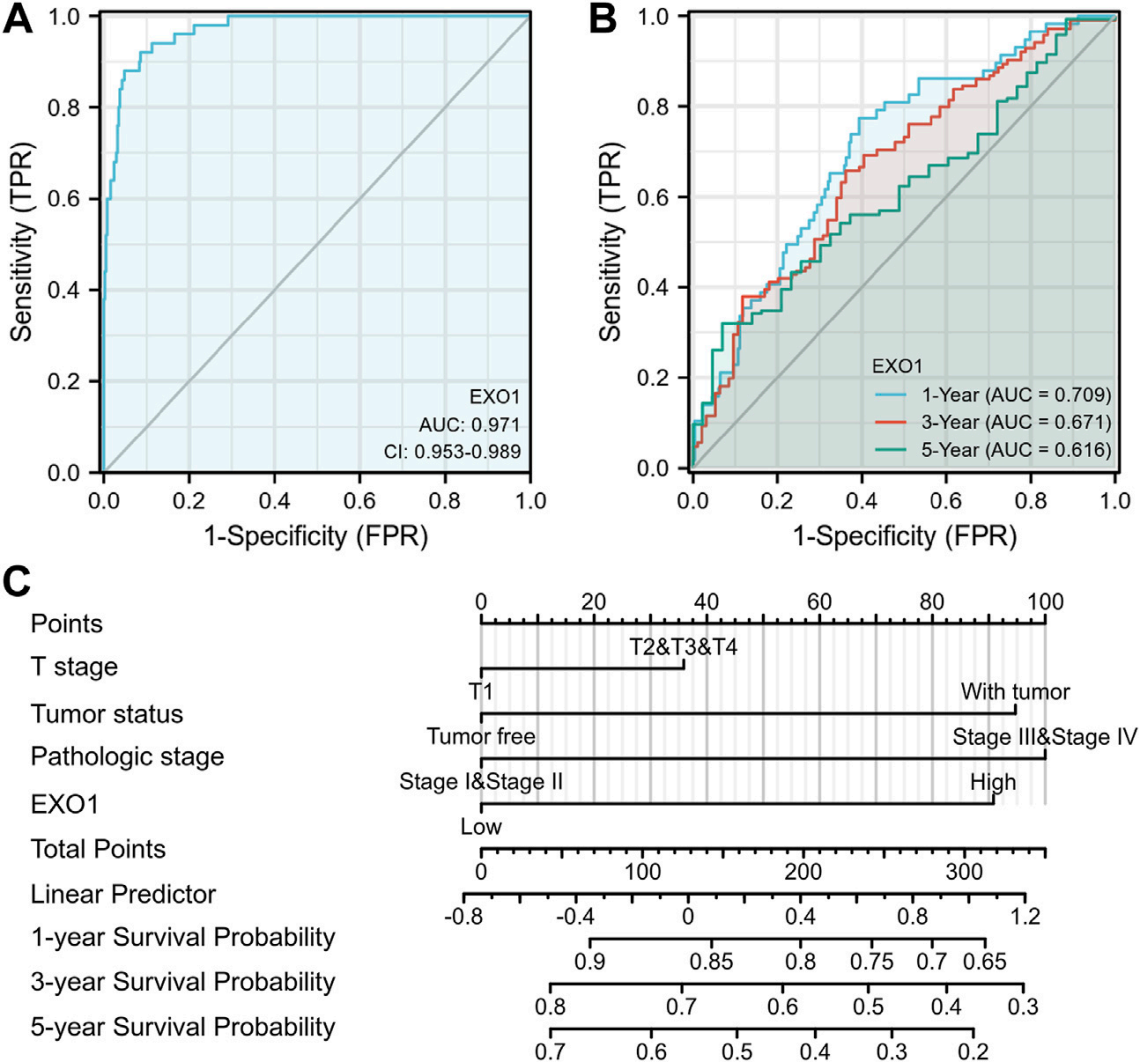
The nomogram model with EXO1 shows a superior diagnostic and prognostic performance in HCC. **(A)** Diagnostic ROC curves to distinguish HCC tissues and normal tissues based on the EXO1 expression levels. **(B)** Time-dependent survival ROC curves to predict 1-, 3-, and 5-year survival rates of HCC patients based on the EXO1 expression levels. **(C)** ROC curve analysis to evaluate the prediction efficacy of the nomogram model that includes clinicopathological factors (T stages, tumor status, and pathologic stages) and EXO1 expression levels to predict the 1-, 3-, and 5-year survival rates of HCC patients.

An ROC curve analysis was performed to determine the diagnostic value of the EXO1 expression levels. The EXO1 expression levels accurately distinguished tumor tissues from the adjacent peritumoral tissues with an AUC value of 0.971 (Figure 9A). The time-dependent ROC curve analysis showed that the AUC values for the predicted 1 -, 3-, and 5-year survival rates of HCC patients based on the EXO1 expression levels were above 0.6 (Figure 9B). A nomogram model was constructed that included T stages, tumor status, pathologic stages, and EXO1 expression levels as parameters. These factors were established as highly significant prognostic prediction factors based on the multivariate Cox regression analysis. The nomogram showed a significantly high clinical value in predicting the 1 -, 3-, and 5-year survival probability of the HCC patients (Figure 9C).

### Prognostic Performance of Exonuclease 1 in the Clinicopathological Subgroups of Hepatocellular Carcinoma Patients

**FIGURE 10:**
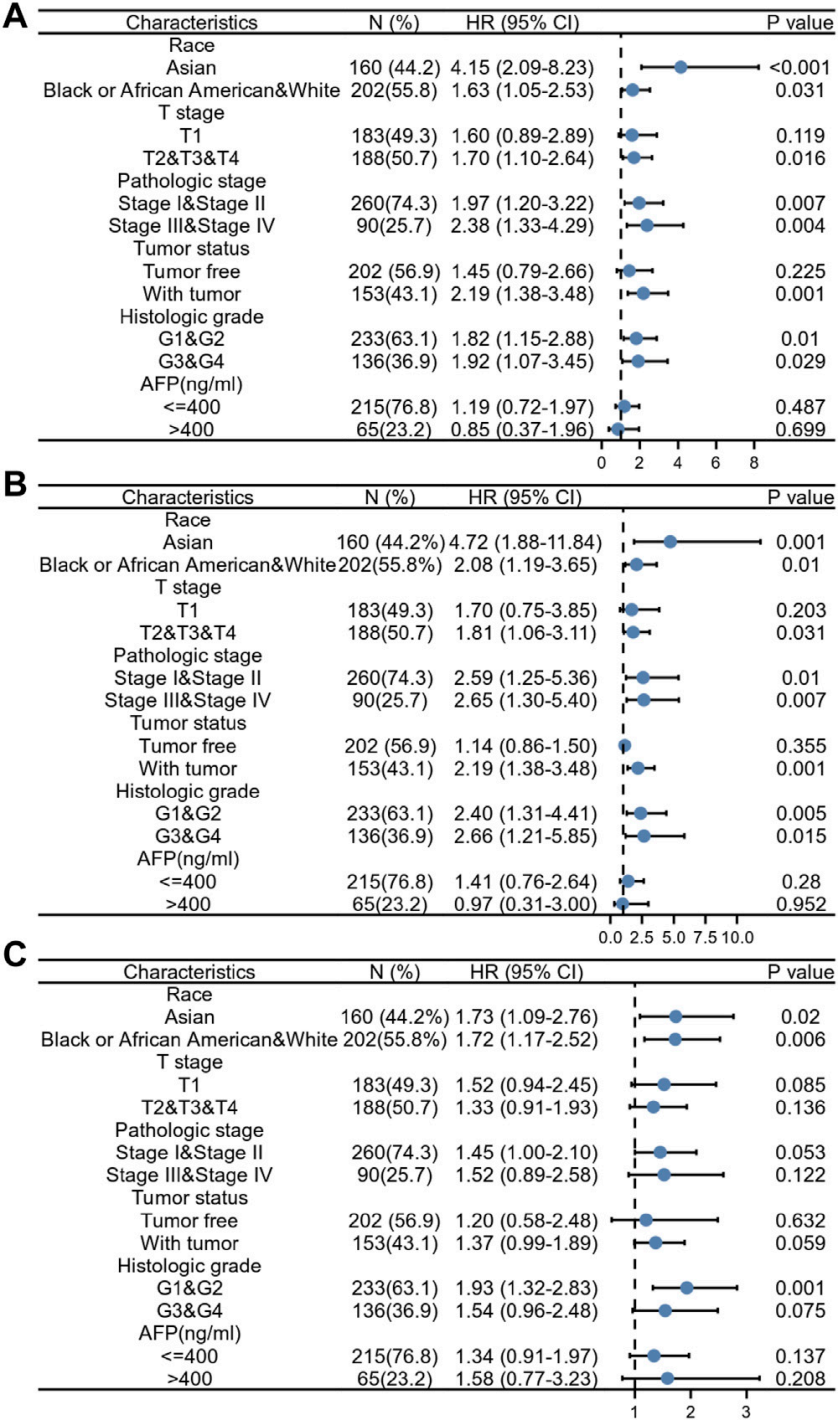
Prognostic performance of EXO1 expression in different HCC patient subgroups. The HCC patients were divided into different subgroups based on race, T stages, pathologic stages, tumor status, histologic grades, and AFP levels. **(A–C)** The Cox regression analysis results show the prognostic performance of EXO1 expression levels regarding **(A)** overall survival, **(B)** disease-specific survival, and **(C)** progression-free interval in different subgroups of HCC patients. The results are represented by the hazard ratio (HR). The bar represents the 95% confidence interval (CI) of the HR values and the size of the diamond represents the significance of the prognostic performance EXO1.

Table 5 and Figure 10 show the results of the Cox regression analysis for specific HCC patient subgroups based on the clinicopathological parameters to determine the predictive value of EXO1. High EXO1 expression levels were associated with unfavorable OS in the HCC patients of different races, especially Asians (HR = 4.15, *p* < 0.001), clinical T2, T3, and T4 stages (HR = 1.70, *p* = 0.016), clinical pathologic stages, tumor status (HR = 2.19, *p* = 0.001), and clinical histologic grades, G1 and G2 (HR = 1.82, *p* = 0.01) (Figure 10A), and DSS (Figure 10B). High EXO1 expression levels were also associated with lower PFI in different races and clinical histologic grades, G1and G2 (HR = 1.93, *p* = 0.001) (Figure 10C). These results demonstrated that the survival rates of HCC patients with a high EXO1 expression were significantly shorter than those with a low EXO1 expression.

**TABLE 5:** Prognostic performance of EXO1 on the clinical outcomes of HCC patient subgroups based on the Cox regression analysis.

Characteristics	N (%)	HR for OS (95% CI)	HR for DSS (95% CI)	HR for PFI (95% CI)
Race				
Asian	160 (44.2)	4.15 (2.09–8.23)^***^	4.72 (1.88–11.84)^***^	1.73 (1.09–2.76)^*^
Black or African American and White	202 (55.8)	1.63 (1.05–2.53)^**^	2.08 (1.19–3.65)^**^	1.72 (1.17–2.52)^**^
T stage				
T1	183 (49.3)	1.60 (0.89–2.89)	1.70 (0.75–3.85)	1.52 (0.94–2.45)
T2&T3&T4	188 (50.7)	1.70 (1.10–2.64)^*^	1.81 (1.06–3.11)^*^	1.33 (0.91–1.93)
Pathologic stage				
Stage I &Stage II	260 (74.3)	1.97 (1.20–3.22)^**^	2.59 (1.25–5.36)^**^	1.45 (1.00–2.10)
Stage III &Stage IV	90 (25.7)	2.38 (1.33–4.29)^**^	2.65 (1.30–5.40)^**^	1.52 (0.89–2.58)
Tumor status				
Tumor free	202 (56.9)	1.45 (0.79–2.66)	1.14 (0.86–1.50)	1.20 (0.58–2.48)
With tumor	153 (43.1)	2.19 (1.38–3.48)^***^	2.19 (1.38–3.48)^***^	1.37 (0.99–1.89)
Histologic grade				
G1&G2	233 (63.1)	1.82 (1.15–2.88)^**^	2.40 (1.31–4.41)^**^	1.93 (1.32–2.83)^***^
G3&G4	136 (36.9)	1.92 (1.07–3.45)^*^	2.66 (1.21–5.85)^*^	1.54 (0.96–2.48)
AFP (ng/ml)				
≤400	215 (76.8)	1.19 (0.72–1.97)	1.41 (0.76–2.64)	1.34 (0.91–1.97)
>400	65 (23.2)	0.85 (0.37–1.96)	0.97 (0.31–3.00)	1.58 (0.77–3.23)

HCC, hepatocellular carcinoma; CI, confidence interval; HR, hazard ratio; OS, overall survival; DSS, disease specific survival; PFI, progress free interval; AFP, alpha-fetoprotein; *p < 0.05; **p < 0.01; ***p < 0.001.

## Discussion

In this study, we demonstrated that EXO1 was significantly overexpressed in 19 out of the 33 human cancer tissues. A high EXO1 expression was observed in the HCC tissues from clinical samples as well as the TCGA and GEO databases. Furthermore, a high EXO1 expression was observed in the serum of HCC patients from the GSE114564 dataset. This suggested that EXO1 was a potential serum biomarker for HCC.

The *in vitro* silencing of EXO1 in the HCC cells reduced cellular proliferation (Dai et al., 2018). Our results showed that EXO1-associated DEGs in the HCC tissues were enriched in biological processes such as organelle fission, nuclear division, chromosome segregation, and nuclear chromosome segregation. EXO1-associated DEGs were enriched in cellular components such as the condensed chromosome and centromeric region as well as molecular functions such as channel activity, motor activity, neurotransmitter receptor activity, and microtubule motor activity. GSEA results showed that HCC tissues with high EXO1 expression were enriched in DEGs related to the cell cycle and DNA damage repair. These data suggested that high EXO1 expression promoted HCC tumorigenesis by regulating cell cycle and DNA damage repair mechanisms.

In a previous study, 25% of HCC samples showed the expression of biomarkers for the inflammatory response (Sia et al., 2017). Our study demonstrated a potential relationship between EXO1 expression and tumor immune cell infiltration. EXO1 expression showed negative correlation with the levels of neutrophils, dendritic cells, and CD8^+^ T cells in the HCC tissues. Neutrophils play a significant anti-tumor role by activating immune responses against the tumor cells and the direct lysis of tumor cells (Eruslanov et al., 2014). Dendritic cells are the most effective antigen-presenting cells that initiate anti-tumor immunity by activating the CD8^+^ T cells (Fu and Jiang, 2018). Our data showed a positive correlation between EXO1 expression levels and the proportion of T follicular helper cells, T helper cells, and type 2 T helper cells in the HCC tissues. T follicular helper cells regulate tumor growth and progression via CXCR5, the chemokine receptor (Breitfeld et al., 2000; Höpken and Rehm, 2012). The transforming growth factor-β (TGB-β) expressed by the regulatory T helper cells (Tregs) and type 2 T helper cells plays a significant role in the resistance mechanisms against cancer immunotherapy (Hong et al., 2008; Li et al., 2020). Furthermore, Th2-derived cytokines such as IL-4 and IL-13 promote tumor progression by inducing M2 macrophage polarization (Biswas and Mantovani, 2010). Therefore, our data suggested that the EXO1 overexpression played a significant role in the immune escape mechanisms of HCC cells, thereby contributing to HCC growth and progression.

CTLA-4 and PDCD-1 are two critical proteins associated with tumor immune escape (Krummel and Allison, 1996; Goodman et al., 2017). Immune checkpoint inhibitors (ICIS) such as ipilimumab (CTLA-4 inhibitor) and nivolumab (PDCD-1 inhibitor) significantly improve the overall survival rates of patients with melanoma (Hodi et al., 2010; Long et al., 2017) and advanced liver cancer (Waidmann, 2018). Furthermore, TP53 is highly expressed in malignant tumors and TP53 mutations are associated with the poor prognosis of several human cancers (Wang and Sun, 2017). TP53 mutations suppressed anti-tumor immunity and reduced the efficacy of cancer immunotherapy (Jiang et al., 2018; Xiao et al., 2018; Lyu et al., 2019). Therefore, we evaluated the relationship between EXO1 expression levels and immune checkpoint genes including CTLA-4 and PDCD-1 as well as TP53. Our results showed positive correlations between the expression levels of EXO1 and the expression levels of immune checkpoint genes and TP53 in the HCC tissues. This suggested that EXO1 was a potential target for improving the efficacy of immunotherapy in HCC patients.

DNA methylation is a common epigenetic mechanism that plays a significant role in tumorigenesis. Changes in the methylation status of several genes have been associated with the initiation, growth, and progression of various cancers (Irizarry et al., 2009; Baylin and Jones, 2011,2016). We investigated the relationship between methylation levels in the *EXO1* gene and the prognosis of HCC patients. The hypermethylation of 5 CpG sites including cg03116938, cg11837910, and cg22022181 were associated with a poor overall survival. These three CpG sites showed the highest degree of DNA methylation. Mutations in the *TP53*, *CTNNB1*, *TERT* promoter, *AXIN1*, *ARID1A*, and *ARID2* genes are associated with the diagnosis and treatment of HCC patients (De Stefano et al., 2018). *TERT* promoter mutations have been reported in the early stages of HCC, whereas *TP53* mutations have been reported in the late tumor stages of HCC (Li et al., 2011; Schulze et al., 2015). Our study showed that the incidence of *EXO1* gene mutations was only 7% in HCC tissues. Furthermore, *EXO1* gene mutations were not associated with OS and DSS in the HCC patients.

Our investigation also showed that EXO1 expression levels in the HCC tissues significantly correlated with the AFP levels, overall survival, disease-specific survival, clinical stages, and the tumor status. Furthermore, higher EXO1 expression levels were associated with a poorer prognosis and advanced clinical stages of the HCC patients. The logistic regression analysis showed that EXO1 expression levels were associated with the clinical stages including T stages and histological stages, tumor status, and AFP levels. K–M survival curves showed that the overall survival, disease-specific survival, and progression-free interval rates of patients with higher EXO1 expression levels were significantly lower than those with lower EXO1 expression levels. The AUC value of EXO1 expression levels for the differential diagnosis of HCC was 0.971. Furthermore, the AUC values of 1-, 3-, and 5-year predicted survival rates were all greater than 0.6. These data suggested that EXO1 was a potential diagnostic and prognostic biomarker for HCC. We also established a nomogram model based on the results of the multivariate Cox regression analysis and showed that EXO1 expression levels significantly improved the prognostic assessment of HCC patients. Previous studies have shown that EXO1 is associated with the poor prognosis of patients with breast cancer (Muthuswami et al., 2013), invasive ductal carcinoma (Kretschmer et al., 2011), and gliomas (de Sousa et al., 2017). These results suggested that EXO1 was a potential prognostic biomarker for multiple cancer types including HCC.

Our study has some limitations. Our results were based on RNA sequencing data of HCC tissues from the TCGA database. However, we could not directly assess the activities of downstream signaling pathways and relative protein levels of EXO1 in the HCC tissues. Therefore, further *in vivo* and *in vitro* experiments are necessary to investigate the mechanisms of EXO1 in HCC.

## Conclusion

In this study, we demonstrated the diagnostic and prognostic values of EXO1 in HCC. EXO1 was overexpressed in the tumor tissues and serum samples of HCC patients. EXO1 regulated HCC progression by modulating the expression of the genes involved in cell cycling and immune response. EXO1 expression levels correlated with tumor infiltration status of many immune cell types and may play a role in the response to immunotherapy in HCC patients. *EXO1* methylation and gene expression was associated with the prognosis of HCC. Therefore, EXO1 is a potential therapeutic target and a useful diagnostic and prognostic biomarker in HCC. However, further studies are needed to validate our findings.

## Data Availability

Publicly available datasets were analyzed in this study. This data can be found here:https://portal.gdc.cancer.gov/(TCGA-LIHC), https://www.ncbi.nlm.nih.gov/geo/ (GSE46408, GSE84402, and GSE114564), https://biit.cs.ut.ee/methsurv/, https://www.cbioportal.org/ (MSK, Clin Cancer Res 2018; AMC Hepatology 2014; and TCGA, Firehose Legacy).
