# Supplementary figures and images for: Exonuclease 1 is a Potential Diagnostic and Prognostic Biomarker in Hepatocellular Carcinoma
Source: Front Mol Biosci. 2022 Jun 13;9:889414. doi: 10.3389/fmolb.2022.889414 (PMC9234278; doi:10.3389/fmolb.2022.889414)

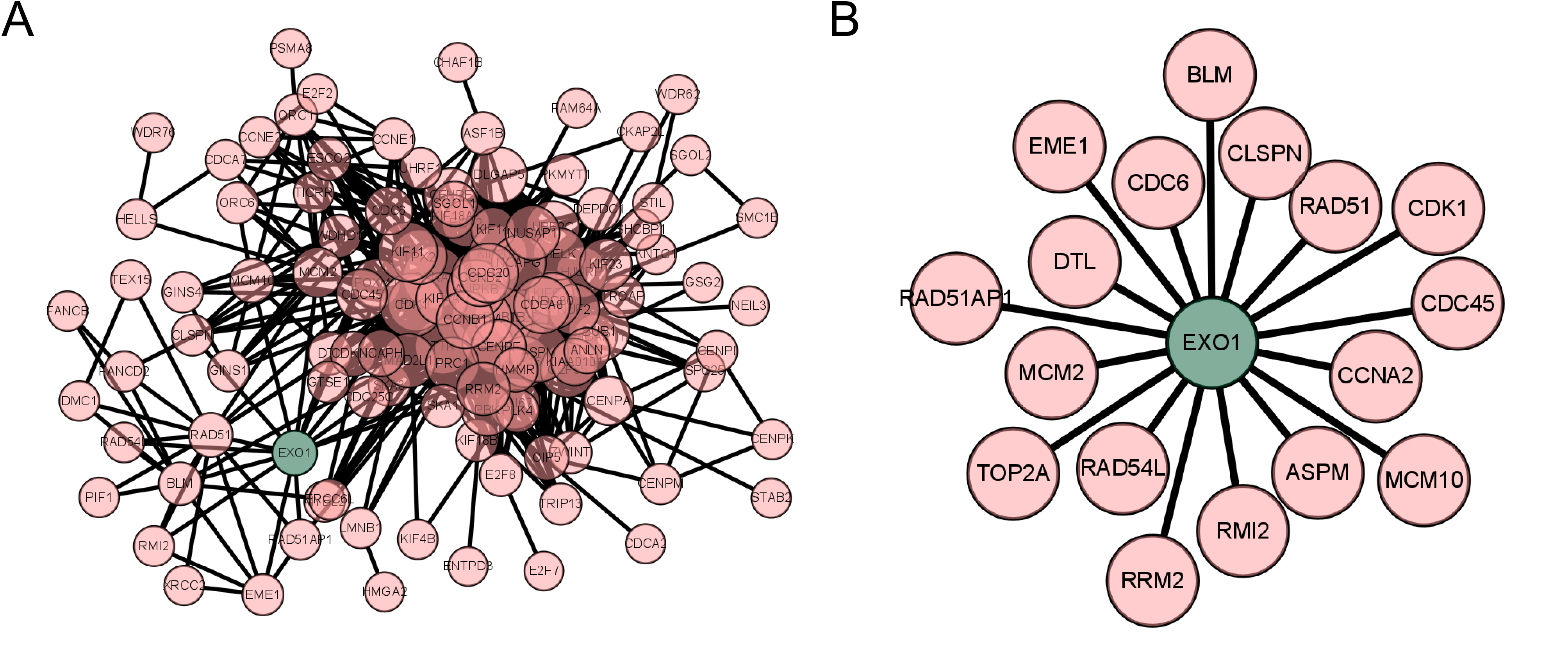

Supplement: Supplementary file 1 [file Image1.TIFF]
