# Supplementary material for: Exonuclease 1 is a Potential Diagnostic and Prognostic Biomarker in Hepatocellular Carcinoma
Source: Front Mol Biosci. 2022 Jun 13;9:889414. doi: 10.3389/fmolb.2022.889414 (PMC9234278; doi:10.3389/fmolb.2022.889414)
Supplement: Supplementary file 2 [file Table1.DOCX]

**Supplementary Figure 1. The protein-protein interaction network of DEGs in HCC patients with different EXO1 levels.** Based on the 794 differentially expressed genes between the high- and low-EXO1 expression groups, we analyzed the interaction using the STRING database, in which the interaction threshold was set to 0.9. Lines represent protein-protein interactions. The larger the circle, the more the interaction between differentially expressed genes **(A)**, and 17 genes directly interact with EXO1 **(B)**.

**Supplementary Table 1. Functional annotations of differentially expressed genes in HCC patients with different EXO1 levels.**

| ONTOLOGY | ID | Description | GeneRatio | BgRatio | pvalue | p.adjust | qvalue |
| --- | --- | --- | --- | --- | --- | --- | --- |
| BP | GO:0007059 | chromosome segregation | 55/682 | 321/18670 | 5.26e-22 | 1.33e-18 | 1.23e-18 |
| BP | GO:0000280 | nuclear division | 62/682 | 407/18670 | 6.63e-22 | 1.33e-18 | 1.23e-18 |
| BP | GO:0098813 | nuclear chromosome segregation | 48/682 | 262/18670 | 1.33e-20 | 1.78e-17 | 1.64e-17 |
| BP | GO:0048285 | organelle fission | 63/682 | 449/18670 | 2.45e-20 | 2.47e-17 | 2.27e-17 |
| BP | GO:0140014 | mitotic nuclear division | 46/682 | 264/18670 | 6.98e-19 | 5.62e-16 | 5.17e-16 |
| CC | GO:0000775 | chromosome, centromeric region | 33/728 | 193/19717 | 1.62e-13 | 7.28e-11 | 6.44e-11 |
| CC | GO:0000776 | kinetochore | 27/728 | 135/19717 | 5.56e-13 | 1.25e-10 | 1.10e-10 |
| CC | GO:0000779 | condensed chromosome, centromeric region | 25/728 | 118/19717 | 1.03e-12 | 1.55e-10 | 1.37e-10 |
| CC | GO:0000793 | condensed chromosome | 34/728 | 223/19717 | 2.07e-12 | 2.32e-10 | 2.05e-10 |
| CC | GO:0000777 | condensed chromosome kinetochore | 23/728 | 105/19717 | 4.01e-12 | 3.60e-10 | 3.18e-10 |
| MF | GO:0003777 | microtubule motor activity | 14/663 | 84/17697 | 2.63e-06 | 0.002 | 0.002 |
| MF | GO:0003774 | motor activity | 17/663 | 136/17697 | 1.33e-05 | 0.005 | 0.004 |
| MF | GO:0030594 | neurotransmitter receptor activity | 15/663 | 117/17697 | 3.14e-05 | 0.007 | 0.006 |
| MF | GO:0015267 | channel activity | 35/663 | 456/17697 | 5.10e-05 | 0.007 | 0.007 |
| MF | GO:0022803 | passive transmembrane transporter activity | 35/663 | 457/17697 | 5.33e-05 | 0.007 | 0.007 |

**Supplementary Table 2. Correlation between EXO1 expression and immune cell biomarkers.**

| Immune cell | Biomarker | Cor | p value |
| --- | --- | --- | --- |
| B cell | CD19 | 0.279 | *** |
|  | CD20 (KRT20) | 0.198 | *** |
|  | CD38 | 0.163 | ** |
| CD8+ T cell | CD8A | 0.185 | *** |
|  | CD8B | 0.172 | *** |
| TFH | BCL6 | 0.169 | *** |
|  | ICOS | 0.306 | *** |
|  | CXCR5 | 0.177 | *** |
| Th1 | T-bet (TBX21) | 0.060 | 0.248 |
|  | STAT1 | 0.403 | *** |
|  | STAT4 | 0.261 | *** |
|  | IL12RB2 | 0.124 | * |
|  | WSX1 (IL27RA) | 0.405 | *** |
|  | IFN-γ (IFNG) | 0.269 | *** |
|  | TNF-a (TNF) | 0.234 | *** |
| Th2 | CCR3 | 0.336 | *** |
|  | GATA3 | 0.148 | ** |
|  | STAT5A | 0.302 | *** |
|  | STAT6 | 0.161 | ** |
| Th9 | IRF4 | 0.176 | *** |
|  | PU.1 (SPI1) | 0.306 | *** |
|  | TGFBR2 | 0.032 | 0.540 |
| Th17 | IL-17A | 0.056 | 0.277 |
|  | IL-21R | 0.305 | *** |
|  | IL-23R | 0.196 | *** |
|  | STAT3 | 0.145 | ** |
| Th22 | AHR | 0.054 | 0.299 |
|  | CCR10 | 0.441 | *** |
| Treg | CCR8 | 0.372 | *** |
|  | CD25 (IL2RA) | 0.273 | *** |
|  | FOXP3 | 0.115 | * |
| M1 macrophage | COX2 (PTGS2) | 0.082 | 0.113 |
|  | INOS (NOS2) | 0.009 | 0.861 |
|  | IRF5 | 0.392 | *** |
| M2 macrophage | ARG1 | -0.157 | ** |
|  | CD206 (MRC1) | 0.074 | 0.154 |
|  | CD115 (CSF1R) | 0.154 | ** |
| TAM | PDCD1LG2 | 0.103 | * |
|  | CD80 | 0.351 | *** |
|  | CD40 | 0.231 | *** |
|  |  |  |  |
